# Case Report: Semaglutide-associated depression: a report of two cases

**DOI:** 10.3389/fpsyt.2023.1238353

**Published:** 2023-08-29

**Authors:** Jia-Rui Li, Jinya Cao, Jing Wei, Wenqi Geng

**Affiliations:** Department of Psychological Medicine, Peking Union Medical College Hospital (CAMS), Beijing, China

**Keywords:** adverse reactions and cases, case report, depression, semaglutide, weight loss

## Abstract

Semaglutide, as a glucagon-like peptide-1 receptor agonist (GLP-1 RA), was approved for glucose control in type 2 diabetes mellitus in 2017 and approved for weight loss in 2021 by the U.S. Food and Drug Administration (FDA). No psychiatric adverse effect associated with semaglutide has been reported so far. Here we report two cases of semaglutide-associated depression. One is a middle-aged man with no previous history of depression who developed depressive symptoms about 1 month after taking semaglutide. The other one is a middle-aged woman with recurrent depressive disorder whose symptoms also recurred about 1 month after semaglutide treatment. Depression was improved or relieved after discontinuation of semaglutide in both cases. Possible psychiatric adverse effects of depression should be taken into consideration when semaglutide is administered to patients.

## 1. Introduction

Glucagon-like peptide-1 receptor agonists (GLP-1 RA) enhance glucose-dependent insulin secretion and exhibit other antihyperglycemic actions. Since the first GLP-1 RA (exenatide twice daily) was approved in 2005 by U.S. Food and Drug Administration (FDA), a series of GLP-1 RAs have been on the market (1). Semaglutide, also a GLP-1 RA, got approval in 2017 by U.S. Food and Drug Administration (FDA)for the treatment of type 2 diabetes mellitus to control blood glucose (initiated at a dose of 0.25 mg once weekly for the first 4 weeks and the dose gradually increased to 1 mg once weekly) (2). Then in 2021, it was approved for weight loss by FDA. Adults with obesity achieved weight loss with once-weekly semaglutide at a dose of 2.4 mg (initiated at a dose of 0.25 mg once weekly for the first 4 weeks, with the dose increased every 4 weeks to reach the maintenance dose of 2.4 mg weekly by week 16 in adjunct to lifestyle intervention) (3).

At present, most reported adverse events of semaglutide were gastrointestinal system discomforts that include nausea, vomiting, diarrhea, and decreased appetite (4–6). It has been associated with severe gastrointestinal adverse events, gallbladder-related disorders, and hepatobiliary disorders (3, 7). There have been also some reports of rare adverse events including hyperglycemia, acute pancreatitis, and acute nephritis (5, 8, 9). In the global phase 3 Semaglutide Treatment Effect in People with Obesity (STEP) program, no difference was found between semaglutide and placebo regarding psychiatric adverse effects (3). However, psychiatric adverse effects, such as suicidal ideation (10), have been reported in studies with other GLP-1 RAs (3, 11).

Here we report two cases of semaglutide-associated depression. One is a middle-aged man with no previous history of depression who developed depressive symptoms about 1 month after taking semaglutide. The other is a middle-aged woman with recurrent depression disorder whose symptoms also recurred about 1 month after semaglutide treatment. Depression was improved or relieved after discontinuation of semaglutide in both cases.

## 2. Case reports

### 2.1. Case 1

On June 1, 2023, a 54-year-old man came to Peking Union Medical College Hospital (Beijing, China) with a complaint of decreased energy and motivation for more than 1 month. His blood glucose level was not monitored after semaglutide treatment, but the patient reported no conspicuous hypoglycemia symptoms, such as hunger, heart palpitations, sweating, etc. The BMI for him is 23.2 kg/m^2^. He is a successful company manager and has had no history of mood disorders. He had been optimistic, active, efficient, and had many interests before this episode of depression. At the end of March 2023, the patient started giving himself injections of semaglutide for weight loss (0.25 mg weekly for the first 4 weeks, then increased to 0.5 mg weekly). Gastrointestinal discomforts (early satiety, abdominal distension, and nausea) were noted shortly after semaglutide injection. About 1 month after starting semaglutide, he began to gradually feel fatigued, not relieved by rest and sleep. At the same time, he developed symptoms of decreased interest and motivation, difficulty concentrating and making decisions, and an increased need for sleep.

As to past medical history, the patient has been diagnosed with hypertension, hyperglycemia, and ankylosing spondylitis for several years, but these conditions are mild and stable. He has not taken any medications for these conditions following the advice of physicians. He had been smoking and drinking in small amounts for decades, but since April his interest in smoking and drinking also diminished, his intake of cigarettes and alcohol decreasing. He has no history of other psychoactive substance use. As he was concerned about the physical and psychological changes, a battery of tests including head MRI and thyroid function was done, and no significant problems were found.

**Figure 1:**
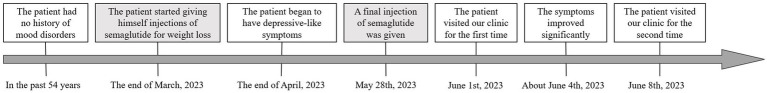
The timeline of case 1.

From mental state examination, mild to moderate depression was established, and the possibility of other psychiatric disorders were ruled out. A possible link between semaglutide and depression was considered. The patient was advised to discontinue semaglutide. No other medication was prescribed. The patient returned to the clinic 1 week later (11 days after the last semaglutide injection) and reported that his symptoms of low mood, difficulty in concentration, fatigue, and hypersomnia were relieved significantly, and he was about 80% of his normal state. Stomach bloating was also reduced. On July 11, the patient returned for a follow-up visit. His mood returned to normal and has resumed full work, family, and social functions. The patient’s feedback was that after stopping semaglutide his condition gradually returned to normal status and no further psychiatric consultation was needed. He expressed satisfaction with the diagnosis and advice he received. The timeline of Case 1 is shown in Figure 1.

### 2.2. Case 2

This is a 40-year-old female patient with recurrent major depressive disorder. She has had three episodes of depression in the last 20 years. The first two episodes each lasted approximately 1 year. She had complete remissions with escitalopram treatment in the first two episodes of depression with no psychotherapy. In 2022, she had a recurrence of depression with similar symptoms lasting almost 1 year. During the worst of the episodes, she experienced intense suicidal ideation and had a profound decrease in motivation and cognitive function. She had been stable since January 2023 on escitalopram 20 mg daily together with psychotherapy. With the treatment described above, her symptoms largely improved, but she had not been able to return to work. As to past medical history, she has been diagnosed with type 1 diabetes for 5 years and has been on insulin, metformin, and voglibose treatment since then. She is also overweight (BMI = 26.81 kg/m^2^) and has insulin resistance.

**Figure 2:**
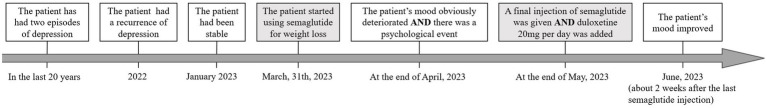
The timeline of case 2.

Her endocrinologist added semaglutide to the diabetic treatment on March 31, 2023. The weekly dosage was 0.25 mg in the first 4 weeks and then increased to 0.5 mg. She had decreased appetite and lost 4 kg of body weight in 2 months after taking semaglutide. The blood glucose was more stable, and the usage of insulin was reduced to 70% of the previous dosage. At the end of April, her mood obviously deteriorated. During the same period, she had some disagreements with her mother. The doctor and the patient agreed to work on this issue, as similar situations had happened before and her condition improved with psychotherapy. But within the next month, her mood deteriorated further. She also developed fatigue, slowing of mind, increased need for sleep, and recurrence of suicidal ideation. The symptoms were similar to previous depressive episodes. From mental state examination, a relapse of severe depression was established. This was unusual considering a past history of positive psychotherapeutic response. At the end of May, semaglutide was stopped for cautious considerations in consultation with her endocrinologist, and duloxetine 20 mg per day was added to escitalopram 20 mg per day. The patient’s mood improved about 2 weeks after the last semaglutide injection. At a follow-up visit on July 18, her condition continued to improve, to which the patient was pleased with the recovery. The patient’s feedback was that her mood has been improving from 2/10 to 5/10 (a subjective feeling of happiness), and her suicidal thoughts have almost disappeared. She continues to worry about enhanced appetite and increased body weight (1 kg in 2 weeks). The timeline of case 2 is shown in Figure 2.

## 3. Discussion

Diabetes and obesity have become main global threats to health in recent decades. In China, the prevalence of overweight and obesity are 34.3 and 16.4%, respectively, (12). According to the World Health Organization, 39% of adults were overweight and about 13% of the world’s adult population were obese in 2016 (13). Approximately 8.5% of adults had diabetes in 2014, and diabetes was the direct cause of 1.5 million deaths and 48% of those deaths occurred before the age of 70 years in 2019 (14). Semaglutide has shown good promise in controlling blood glucose and reducing body weight. Although no psychiatric adverse effects of semaglutide were reported up to now, semaglutide has only been widely used in clinics for a relatively short time. Therefore, its efficacy and safety in the real world still need to be further observed. In the present case report, the occurrence or recurrence of depression was observed about 1 month after semaglutide use. After discontinuation of semaglutide, depression significantly improved. This suggests that semaglutide therapy may cause psychiatric adverse effect of depression in some patients.

In Case 1, the patient developed a lack of interest in smoking and alcohol drinking after semaglutide injection. Studies in rodents and non-human primates have demonstrated that GLP-1 RAs can lead to a reduction in the intake of alcohol and drugs of abuse (15). As to semaglutide, it reduced ethanol preference on the day of injection in rats but the effect was transient, lasting no longer than 48 h (16). No rodents or clinical research about the inhibition of the desire for nicotine has been reported with semaglutide.

In Case 2, the depression could be related to a combination of factors. In addition to semaglutide, other factors, such as stress related to the diagnosis and prognosis of diabetes, and psychological events may also have contributed to her current episode. But these factors have been relatively chronic and were not commensurate with the sudden worsening of depression. The unresponsiveness to psychotherapy, in contrast to past treatment experience, also suggests there may be some new contributing factors. The patient’s symptoms improved considerably 2 weeks after discontinuation of semaglutide. While the added effect of 20 mg duloxetine per day may have been contributing to this improvement, based on clinical experience, this effect would be relatively slow and gradual. Therefore, the possible effect of semaglutide discontinuation cannot be neglected.

GLP-1 is a hormone that inhibits glucagon secretion and promotes insulin secretion (17). GLP-1 RAs have been shown good promise in the treatment of type 2 diabetes and can also induce weight loss, which is thought to be related to its peripheral effect of delaying gastric emptying and its central anorexigenic effects (4, 18). As a long acting GLP-1 RA, semaglutide can translocate to the brain stem, lateral septum (LS), and hypothalamus, interact directly with various GLP-1 receptor subtypes and modulate the activity of neural pathways involved in food intake, reward, and energy expenditure (19, 20). GLP-1 receptor stimulation can increase dopaminergic neuron activity via a presynaptic mechanism of action in the ventral tegmental area (VTA) (21) and increase the expression of dopamine transporter on the neuronal cell surface to reduce free dopamine levels in the synapses in LS and striatal brain (22, 23). Dopamine plays a prominent role in the immediate reinforcing/rewarding effects, and the dysfunction of reward system is an important mechanism of anhedonia and other depressive symptoms (24).

Further case registration studies are needed to clarify the relationship between semaglutide and depression. Endocrinologists and psychiatrists should both pay attention to patients’ emotional status before and after semaglutide use. The mechanism of the possible causality between semaglutide and depression is still unclear. Further research may help to improve understanding of the mechanism of depression and even lead to new therapeutic targets of depression.

Both patients gave their consent to the publication of their cases.

## Data availability statement

The original contributions presented in the study are included in the article/supplementary material, further inquiries can be directed to the corresponding author.

## Ethics statement

The studies involving humans were approved by the Ethical Committee of Peking Union Medical College Hospital. The studies were conducted in accordance with the local legislation and institutional requirements. The participants provided their written informed consent to participate in this study.

## Author contributions

J-RL mainly participated in literature study and article writing. JC participated in article writing, provided case information, and research direction guidance. JW provided case information and guided research direction. All authors contributed to the article and approved the submitted version.

## Funding

This work was supported by the National High Level Hospital Clinical Research Funding (Grant Number: 2022-PUMCH-B-093) and the STI2030-Major Projects (Grant Number: 2021ZD0202001). Funders played no role in the content of this manuscript.

## Conflict of interest

The authors declare that the research was conducted in the absence of any commercial or financial relationships that could be construed as a potential conflict of interest.

## Publisher’s note

All claims expressed in this article are solely those of the authors and do not necessarily represent those of their affiliated organizations, or those of the publisher, the editors and the reviewers. Any product that may be evaluated in this article, or claim that may be made by its manufacturer, is not guaranteed or endorsed by the publisher.
